# Factors Affecting HIV Testing among Youth in Kenya

**DOI:** 10.3390/ijerph16081450

**Published:** 2019-04-24

**Authors:** Allison Nall, Tiffany Chenneville, Lindsey M. Rodriguez, Jennifer L. O’Brien

**Affiliations:** Psychology Department, College of Arts and Sciences, University of South Florida St. Petersburg, St. Petersburg, FL 33701, USA; anall@mail.usf.edu (A.N.); lrodriguez12@mail.usf.edu (L.M.R.); jenobrien@usf.edu (J.L.O.)

**Keywords:** HIV, AIDS, testing, social cognitive theory, youth, Kenya

## Abstract

With the high prevalence of HIV among youth in sub-Saharan Africa, it is vital to better understand factors affecting HIV testing among this population; this is the first step in the HIV treatment cascade. The purpose of this study was to examine factors related to behavioral intentions regarding HIV testing using existing pre-test data from the HIV SEERs (Stigma-reduction via Education, Empowerment, and Research) Project, a community-based participatory research program targeting 13–24-year-olds in Kenya. It was hypothesized that HIV knowledge, social support, subjective well-being, and mental health (depression, anxiety, and stress) would serve as facilitators to HIV testing while projected stigma and substance use would serve as barriers to HIV testing. In partial support of our hypotheses, findings from logistic regression analyses revealed that HIV knowledge, substance use, depression, and social support were significant predictors of HIV testing intentions. However, HIV knowledge and substance use served as facilitators while depression and social support served as barriers. While projected stigma was correlated with HIV testing intentions, it was not a significant predictor in the regression analysis. Subjective well-being, anxiety, and stress were not significant predictors in the regression analysis. These findings have important implications for HIV testing initiatives designed for youth in Kenya as well as future research on HIV testing with this population.

## 1. Introduction

The Joint United Nations Programme on HIV/AIDS [1] estimated the number of youth (15–24-years old) living with HIV as of 2017 to be 3.9 million worldwide. Among the global population of youth between the ages 15–24, there were an estimated 610,000 new cases in 2016 according to the United Nations Children’s Fund [2]. Approximately 84% of these new cases occurred in Sub-Saharan Africa [2]. These numbers suggest a need to further investigate HIV prevention efforts among youth, especially in parts of the world with high HIV prevalence rates. Sub-Saharan Africa accounts for approximately 64% of all new HIV diagnoses worldwide [3]. Of the 630,000 children being administered antiretroviral therapy in countries designated as low-and middle-income, 544,000 live in Africa according to the World Health Organization [4]. This population faces many challenges associated with health services, including stigma, lack of prevention education, and lack of support services and treatment options [1].

### 1.1. HIV Testing as An HIV Prevention Strategy

HIV testing is a vital step for both treatment and prevention [5]. Once aware of HIV infection, patients can begin antiretroviral therapy (ART), which is designed to reduce viral load. Current research suggests people with HIV who have undetectable viral loads are not likely to transmit the virus to others. Thus, identifying cases of HIV through testing and linking newly diagnosed patients to, and retaining them in, care has been shown to be effective for preventing the progression of the virus and eventual spread to others. 

According to the Kenya HIV Testing Services Guidelines from the National AIDS and Sexually Transmitted Infections (STI) Control Programme [6], all healthcare facilities should offer HIV testing and counseling to adults and adolescents 15 years or older. It is recommended that children 14 years and younger be tested if living in an area identified as having a high incidence of HIV or if the child has been exposed to high-risk situations or environments (e.g., sex work or those whose mothers died from AIDS or for unknown reasons). Nonetheless, recent estimates suggest only 46% of women and 53% of men in Kenya’s general population had been tested for HIV, suggesting a need to better understand the facilitators and barriers to HIV testing [7]. 

### 1.2. Facilitators and Barriers to HIV Testing

Barriers to HIV testing exist particularly in areas of high incidence, such as Sub-Saharan Africa. Existing research suggests facilitators to HIV testing may include high levels of knowledge, social support, subjective well-being, and mental health symptoms (e.g., depression, anxiety, stress), while barriers may include high levels of perceived stigma and substance use. In the sections to follow, both facilitators and barriers to HIV testing are explored. 

#### 1.2.1. HIV Knowledge

Behavioral intentions related to HIV testing may be affected by misconceptions about HIV and how the virus is transmitted [8]. Understanding an individual’s HIV knowledge-level is tantamount to understanding how much they perceive risk when engaging in behaviors that could lead to transmission of the virus, such as unprotected sex [9]. Glick and Sahn [9] reported a positive relationship between education and HIV testing in nine African countries. Other research indicates a positive relationship between HIV knowledge and testing [10]. Findings from a meta-analysis of 60 studies revealed that HIV knowledge ranked among the most common factors selected by researchers when studying HIV testing behaviors, and that HIV knowledge was positively correlated with HIV testing [11].

#### 1.2.2. Stigma

HIV-related stigma has been established as a key barrier to HIV testing and treatment [8]. HIV-related stigma can manifest in various forms and be either perceived, anticipated, or believed to be true by an individual or group, notwithstanding HIV status [12]. Simply put, regardless of whether or not an individual has been tested and is aware of their HIV status, one may hold certain beliefs or preconceptions about individuals with HIV. These beliefs often serve as a barrier to both HIV testing and treatment. 

One method being considered as a means to lower stigma related to HIV and HIV testing is to normalize screening for the virus by making screening routine in healthcare settings. In interviews with men aged 18–24, Knight, Small, and Shoveller [13] found that while, overall, participants viewed routine (as opposed to voluntary) testing as more convenient and less stress-inducing than seeking out testing as a result of high-risk behavior, others felt targeted by routine testing. The latter group noted that appearing to be homosexual, a drug user, or impoverished may have led the clinician to recommend testing. The authors concluded that while routine testing may break down certain barriers, making those more at risk feel targeted may be a drawback. 

In a re-analysis of a 2009 study from South Africa, Maughan-Brown and Nyblade [14] found that women (but not men) were influenced by stigma when it came to HIV testing. Further analysis of this dataset showed that women were more likely to have been tested for HIV if they had stigmatizing attitudes toward others (e.g., believed that those with HIV were being punished for having multiple sexual partners and had themselves to blame). Conversely, women who reported more perceived stigma, or had personally observed someone with HIV being treated poorly, were less likely to seek testing. This research highlights the importance of better understanding the influence of stigma on HIV testing behaviors. 

#### 1.2.3. Social Support

Social support has also been identified as a key factor in HIV test-seeking and treatment adherence [8]. Social support can be conceptualized as the notion of having other individuals to provide reinforcement and emotional support [15]. Among nearly 5000 adults in South Africa voluntarily seeking HIV testing and counseling, low social support and poor mental health were associated with testing positively for HIV [16]. When examining correlates of social support, researchers found that low social support was most strongly correlated with having no prior HIV testing [15]. 

Lypen, Lockwood, Shalabi, Harper and Ngugi [17] studied type and source of social support among Kenyans aged 18–27. Their research indicated the important role of social support in populations with high risk of HIV in seeking testing and treatment. However, they stress that many pathways of social support have not yet been explored in HIV intervention studies. Other studies also suggest social support is important for HIV testing and that low levels of social support may serve as a barrier to testing among numerous populations of men who have sex with men (MSM) in the United Kingdom and United States [18,19]. Social support also has been found to be an important factor in disclosing one’s status to others. In their meta-analysis of 21 studies on social support, HIV stigma, and disclosure, Smith, Rossetto, and Peterson [15] found that disclosing one’s positive HIV diagnosis was positively correlated with having some form of social support.

#### 1.2.4. Subjective Well-being

Subjective well-being refers to one’s assessment of tangible and intangible evidence of health or quality of life [20]. Among South Africans, subjective well-being was associated with income and social support from family and religious affiliations. Compared to people without HIV, subjective well-being was significantly lower among people living with AIDS in Australia and the United States [21]. These authors reported a negative correlation between stigma and subjective well-being among people living with HIV.

Currently there is no research on the relationship between subjective well-being and HIV testing. However, findings from a study in Portugal suggested subjective well-being is correlated with adherence to antiretroviral medication, indicating its importance in the scope of treatment and living with HIV [22]. In their study, Reis, Guerra, and Lencastre [22] found that patients with greater subjective well-being were more likely to be asymptomatic. Given its impact on how people living with HIV cope and adhere to medication regimens, subjective well-being is a factor of interest among the HIV research community. 

#### 1.2.5. Mental Health

Mental health symptoms such as depression, anxiety, and stress can affect HIV risk exposure as well as HIV testing and treatment [8]. Among a sample of 325 men and women in Uganda, depression was correlated with alcohol consumption and intimate partner violence—both risk factors for exposure to HIV [23]. In South Africa, researchers conducted interviews based on the Diagnostic and Statistical Manual of Mental Disorders (DSM-5) with patients at HIV testing sites and found that 14% of those seeking testing experienced depression, 5% experienced anxiety, and nearly 5% experienced posttraumatic stress disorder [24]. In India, those seeking repeat voluntary testing were found to have significant levels of depression and anxiety [25]. In a Taiwanese study, researchers found that nearly 40% of their sample of men seeking HIV testing reported depression [26].

The relationship between HIV testing and mental health symptoms (e.g., anxiety, depression, stress) may be bidirectional. That is, individuals with mental health symptoms may be at high risk for exposure to HIV and, therefore, more likely to seek testing at the same time that HIV testing may produce symptoms of anxiety, depression, or stress. For example, Worthington and Meyers [27] identified four themes related to anxiety among individuals seeking HIV testing in Canada: risk to health, stigma, the power dynamic of the patient and test provider, and techniques employed to improve patient control when interacting with test providers. In another study, mothers in rural Kenya reported they may be less likely to take measures to prevent transferring HIV to their infants due to stigma and stress associated with HIV, testing their infants, and seeking drugs to prevent transmission [28]. 

Yehia and colleagues [29] found that individuals with mental illness in the United States were more likely to seek HIV testing than those without a diagnosed mental illness. This research included disorders such as schizophrenia, bipolar disorder, depression, and anxiety, and participants within each category were found more likely to have been tested for HIV than the general population. 

#### 1.2.6. Substance Use

Substance use contributes to HIV on multiple levels; it may affect HIV testing, put individuals at higher risk of contracting the disease, and lower the ability to adhere to treatment regimens [8]. Substance use has been established as a factor that increases HIV risk through engagement in high-risk sexual behavior (e.g., one-night stands, sex without a condom, multiple partners, sharing needles) [30,31]. UNAIDS [32] estimates that individuals who use injection drugs are 28 times more likely to be living with HIV. Substance users in Indonesia noted stigma and fear of test results as the major barriers to their seeking HIV testing [33].

### 1.3. Social Cognitive Theory Applied to HIV

Bandura’s [34] social cognitive theory (SCT) posits that the person, the environment, and one’s behavior equally share multidirectional influence over one’s learning. SCT has often been applied to health behaviors since these are typically learned throughout an individual’s lifetime [35]. Bandura [35] himself contributed to the HIV/AIDS literature by applying SCT to HIV/AIDS education and prevention. Based on a thorough review of the HIV/AIDS literature, Bandura posited that personal factors (specifically self-efficacy), one’s environment, and one’s behavior are constantly interfacing in a way that influences an individual’s decision to engage in risky sexual behavior or to consider HIV testing. Bandura stressed that while understanding HIV risk behaviors is important, the impact of one’s environment and behavioral changes involved in making sound decisions about HIV prevention are also important. Since Bandura’s theory emphasizes a multidirectional interaction between factors, it cannot be assumed knowledge alone will influence health behaviors. Researchers in China applied SCT to HIV education in high schools and found that students who were exposed to an intervention program encompassing self-efficacy, one’s environment, and behavior significantly differed from students in a control group [36] The intervention program focused on HIV knowledge, perceptions, stigma toward those with HIV/AIDS, and behavioral intentions related to testing. 

### 1.4. Purpose of This Study 

Guided by SCT, this study, which was part of a larger research program on HIV-stigma reduction, aimed to examine the relative impact of the following factors, which can be conceptualized as facilitators and barriers, on behavioral intentions related to HIV testing: HIV knowledge, stigma, social support, subjective well-being, mental health, and substance use. Specifically, this study aimed to answer the following question: What is the relative contribution of various factors (HIV knowledge, stigma, social support, subjective well-being, mental health, substance use) on behavioral intentions related to HIV testing among youth in Kenya? We hypothesized that (a) participants who reported greater HIV knowledge, social support, subjective well-being and depression/anxiety/stress (subscales of mental health) would be more likely to seek HIV testing (facilitators) and (b) participants who reported greater HIV projected stigma and substance use would be less likely to seek HIV testing (barriers). 

## 2. Materials and Methods

### 2.1. Study Design 

A one group correlational research design was employed in this study using existing pre-test data from the HIV SEERs Project, a community-based participatory research project involving four components (information, skills building, resources, and personal contact) that was delivered to 1526 participants aged 12–36 in Kenya [37,38,39]. A correlational design is advantageous in the current study because it can help elucidate the strength and direction of relationships between personal/environmental factors and an individual’s intent to receive HIV testing in advance of any intervention. 

### 2.2. Research Variables

Framed by SCT, this study examined the relative contribution of personal, behavioral, and environmental factors affecting HIV testing among youth. Personal factors included HIV knowledge, HIV projected stigma, subjective well-being, mental health and substance use. The environmental factor was social support, and the behavioral factor was HIV testing intent. These factors were conceptualized as either facilitators (HIV knowledge, social support, subjective well-being, depression, anxiety, stress) or barriers (HIV stigma, substance use) to HIV testing. 

### 2.3. Participants and Procedure 

The entire HIV SEERs Project was evaluated using pre, post, and three-month follow-up questionnaires, which were translated using a back-translation method and offered to participants in either English or Swahili, the native language of Kenya. All participants elected to complete the questionnaires in English, which is the language of instruction in Kenya [40]. This study used existing pre-test data only and included 1007 Kenyan youth aged 13–24 who were not HIV-positive. However, 356 participants did not provide complete data on demographic or study-related variables and were therefore not included in the primary analyses. Results henceforth are based on the 651 participants who provided complete data. Participants who were included were not different than participants who were excluded on a number of demographic characteristics: age (*t*(1005) = −1.20, *p* = 0.23), gender (*χ*^2^(1) = 0.763 *p* = 0.38), sexual orientation (*χ*^2^(2) = 2.19, *p* = 0.334), ever having been tested for HIV (*χ*^2^(2) = 0.510, *p* = 0.775), or sexually active status (*χ*^2^(2) = 2.883, *p* = 0.237). The Institutional Review Board at the University of South Florida reviewed this project and deemed it exempt given its use of existing pre-test data from the HIV SEERs Project. 

### 2.4. Measures

A post-hoc analysis of reading level revealed that the Flesch-Kincaid reading level of the measures described below ranged from 4.7 to 7.3 with the exception of the CRAFFT Screening Tool for Adolescent Substance Use, which had a reading level of 9.1.

#### 2.4.1. Behavioral Intentions

This scale was developed by the HIV SEERs Project investigators to collect information about HIV treatment-seeking behaviors. Only one item addressing HIV testing behavioral intentions was used in this study: “If you are not HIV-positive, do you plan to be tested for HIV?” Response options included “Yes,” “No,” and, “I am HIV-positive.” Participants who responded “I am HIV-positive” were excluded from the analysis. Since this measure consisted of a single item, Cronbach’s alpha was not calculated. 

#### 2.4.2. Brief HIV Knowledge Questionnaire (HIV KQ-18)

The HIV KQ-18 [41] is an 18-item instrument that uses true/false prompts to measure an individual’s understanding of HIV and methods of transmission. “A person will NOT get HIV if she or he is taking antibiotics” is an example of the type of items on this measure. Carey and Schroder [41] reported a Cronbach’s α = 0.75 to 0.89 across multiple samples, demonstrating the internal consistency of this measure. In the current sample, α = 0.39 was found. Missing data on this measure was scored as an incorrect. 

#### 2.4.3. AIDS-Related Stigma Scale (ARSS)

The ARSS [42] is a nine-item instrument that assesses an individual’s beliefs and projected stigma toward people living with HIV/AIDS. Response choices include Agree or Disagree. Examples of items on this measure are “People who have AIDSs are dirty,” and “People who have HIV should be isolated.” Kalichman and colleagues [42] reported a Cronbach’s alpha of α = 0.75, demonstrating the internal consistency of this measure across five African communities, and also used test-retest reliability estimates to demonstrate the stability of this measure over a three-month period, *r* = 0.67. In the current sample, α = 0.59 was found. 

#### 2.4.4. Social Provision Scale

The original 24-item measure [43] was adapted to a 12-item measure, which assesses social support using six provisions (attachment, social integration, reassurance of worth, reliable alliance, guidance, and opportunity for nurturance) [44]. Participants respond to items such as “There are people I can depend on to help me if I really need it” using a five-point Likert scale with 1 = Strongly Disagree and 5 = Strongly Agree. In the original sample, α = 0.92 was reported for the entire scale, demonstrating good internal reliability. In the current sample, α = 0.62 was found. 

#### 2.4.5. Subjective Well-being Scale

The Subjective Well-being Scale [45] gauges well-being on a ten-point Likert scale with 1 = Extremely Unhappy and 10 = Extremely Happy using a single question, “Taking all things together, how happy are you?”. Since this measure consisted of a single item, Cronbach’s alpha was not calculated. 

#### 2.4.6. Depression, Anxiety, and Stress Scale (DASS-21)

The DASS-21 [46] is a 21-item measure that assesses depression, anxiety, and stress (the three subscales of the measure). Examples of items from this measure include “I felt scared without any good reason” and “I felt I was close to panic.” Participants respond using a four-point Likert scale where 0 = Did not apply to me at all and 3 = Applied to me very much or most of the time. Cronbach’s alphas ranging from α = 0.87 to α = 0.94 across the three subscales have been reported, demonstrating good internal reliability [47]. In the current sample, α = 0.86 for the depression scale, α = 0.83 for the anxiety, and α = 0.87 for the stress scale. 

#### 2.4.7. CRAFFT Screening Tool for Adolescent Substance Abuse

The CRAFFT [48] is a nine-item, yes/no measure of an individual’s experiences with drugs and alcohol. The assessment is divided into two sections: three behaviors within the last 12 months, and six behaviors over the lifetime. The six lifetime items are used for scoring purposes. Example items from this measure are “During the past 12 months, did you smoke any marijuana or hashish?” and “Do you ever forget things you did while using alcohol or drugs?” Knight and colleagues [48] reported α = 0.79, demonstrating good overall reliability of this measure. In the current sample, α = 0.63 was found for the last six items. 

### 2.5. Date Analysis

Descriptive statistics were used to assess the sample and performance on measures. Cronbach’s alpha was computed to determine the reliability of measures as reported above. Pearson’s *r* was used to examine the bivariate relationship between two continuous variables and point-biserial correlations were used to examine bivariate relationships between a continuous and dichotomous variable. Logistic regression was used to determine the predictive effect of each of the independent variables (i.e., HIV knowledge, stigma, social support, subjective well-being, depression, anxiety, stress, and substance use) on HIV testing intent (1 = yes or 0 = no). Forward selection was used to identify the best combination of independent variables likely to predict HIV testing intentions. We followed suggestions by Lee and Koval [49] and set our alpha level to 0.15 for entry into the analysis. Sexual activity (1= sexually active or 0 = not sexually active) was included as a covariate and tested as a moderator in the analysis. SPSS version 25 (SPSS Inc., Chicago, IL, USA) was utilized to complete all analyses.

## 3. Results

### 3.1. Participant Demographics

Among the 651 participants aged 13–24, the mean age was 16.7 (SD = 3.07). The sample was 53.5% female and 46.5% male. Only 3.5% identified as gay or lesbian while 92.5% did not identify as gay or lesbian and 4.0% wished not to answer. The majority of participants identified their religion as Christianity (95.5%) while 2.9% identified as Islam and 1.4% combined identified as Hindu, Buddhist, or Other. Less than 1% selected No Religion or wished not to answer. Most said they were not sexually active (72.0%) while 20.8% said they were sexually active and 7.2% wished not to answer. A slight majority of the sample (59.3%) reported having been tested for HIV while 40.1% had never been tested and less than 1% wished not to answer.

### 3.2. Descriptive Statistics

#### 3.2.1. Behavioral Intentions 

Approximately two-thirds of participants (67.0%) reported they intended to be tested for HIV, while 33.0% said they did not. 

#### 3.2.2. Brief HIV Knowledge Questionnaire (HIV KQ-18)

Scores on this measure ranged from 4–18 with a mean score of 13.65 (SD = 2.60), suggesting a moderate level of HIV knowledge among participants in this sample. Some common incorrect items on this measure were “Coughing and sneezing DO NOT spread HIV” and “A person can get HIV by sharing a glass of water with someone who has HIV.” 

#### 3.2.3. AIDS-Related Stigma Scale (ARSS)

Scores on the ARSS ranged from 0–10 with a mean score of 1.32 (SD = 1.27), indicating low levels of projected HIV stigma among participants. 

#### 3.2.4. Social Provision Scale

Scores on this scale ranged from 6–60 with a mean score of 34.00 (SD = 7.03), indicating a low to moderate level of perceived social support among the sample. 

#### 3.2.5. Subjective Well-being Scale 

Scores on this scale ranged from 1–10 with a mean score of 7.92 (SD = 2.48) indicating moderate to high levels of reported well-being among the sample. 

#### 3.2.6. Depression, Anxiety, and Stress Scale (DASS-21)

This scale was divided into the three mental health subscales of depression, anxiety, and stress for more valuable interpretation. Depression scores ranged from 0–38 points with a mean of 7.57 (SD = 9.23), indicating low to moderate levels of depression among the sample. Anxiety scores ranged from 0–34 points with a mean of 6.80 (SD = 8.21), indicating low to moderate levels of anxiety reported by the sample. Stress scores ranged from 0–38 points with a mean of 8.08 (SD = 9.40), indicating low to moderate levels of stress reported by participants.

#### 3.2.7. CRAFFT Screening Tool for Adolescent Substance Abuse (CRAFFT)

Participants (*n* = 871) reported total scores ranging from 0-6 with a mean score of 1.39 (SD = 0.81); 30.4% of participants scored two or higher on this measure, which is the recommended threshold for intervention [45].

### 3.3. Impact of Independent Variables on Intent to Test for HIV

We first examined zero-order correlations among all of the study variables. As expected, HIV knowledge was significantly positively related with an intent to seek HIV testing and projected stigma was significantly negatively related to an intent to seek HIV testing. Contrary to expectations, substance use was positively—and social support negatively—correlated with an intent to seek HIV testing. 

We next evaluated our primary hypotheses with a logistic regression model which included intent to be tested for HIV (1 = yes or 0 = no) as the outcome variable and HIV knowledge, stigma, social support, subjective well-being, depression, anxiety, stress, and substance use included as potential predictor variables. Sexual activity was included as a covariate. The logistic regression model was statistically significant, χ^2^(4) = 24.02, *p* < 0.001. The model explained 5.7% (Nagelkerke *R^2^*) of the variance in HIV testing intentions and correctly classified 66% of cases. 

Results from the logistic regression analysis are presented in Table 1. Four variables emerged as significant or marginally significant predictors of HIV intentions: HIV knowledge, substance use, depression, and social support. HIV knowledge was significantly associated with a higher likelihood of intent to seek testing, *b* = 0.087, χ^2^ = 6.19, *p* = 0.013. The odds ratio was 1.09, suggesting that every one-unit increase in HIV knowledge resulted in an 9% increase in HIV testing intentions, reflecting a small effect. Substance use also predicted intent to test for HIV, *b* = 0.346, χ^2^ = 7.33, *p* = 0.007. The odds ratio was 1.41, indicating that a one-unit increase in CRAFFT scores increased intent to test by 41%, reflecting a medium effect. Depression was marginally associated with a lower intent to seek testing, *b* = −0.019, χ^2^ = 3.72, *p* = 0.054, reflecting a small effect. Finally, social support was marginally negatively associated with intent to seek HIV testing, *b* = −0.024, χ^2^ = 3.68, *p* = 0.055, reflecting a small effect. Projected stigma, subjective well-being, anxiety, stress, and sexual activity were not associated with HIV testing intentions and were therefore not included in the final model. Sexual activity did not moderate any of the associations between the predictor variables and intent to test.

## 4. Discussion

Framed within the context of social cognitive theory (SCT), this study used existing data from the HIV SEERS Project, a community-based participatory research project designed to increase HIV knowledge and decrease HIV stigma, to examine facilitators and barriers to HIV testing among youth in Kenya. Understanding what might motivate or prevent Kenyan youth from seeking HIV testing is key in advancing effective education programs for this high-risk population. 

In this study, HIV knowledge and substance use were found to be significant facilitators to HIV testing. The association between HIV knowledge and intent to be tested for HIV is consistent with existing studies [8,9,10,11]. The association between substance use and HIV testing is consistent with some studies, but not others. Similar to findings in our study, Luseno & Wechsberg [50] reported an association between substance use and HIV testing, which may be a function of those using substances perceiving themselves to be at higher risk for HIV. Inconsistent with our findings, Altice, Kamarulzaman, Soriano, Schechter, and Freidland [51] reported that people who used drugs of any kind were less likely to seek HIV testing early on and, for this reason, were more likely to be diagnosed with advanced stages of HIV. Also among their findings were other important health indicators, such as increased risk of HIV exposure and suboptimal healthcare. Given the relationship between HIV testing and substance use, some current research suggests carrying out HIV testing at substance use treatment facilities [52]. Substance use is understood to increase risk-taking behaviors such as unprotected sex, needle sharing, and one-night stands, which may increase the chances of individuals contracting the disease [30,31]. 

Regarding mental health, our results suggested that depression was marginally significantly predictive of intent to test while anxiety and stress did not significantly predict HIV testing. Rueda and colleagues [8] found that depression, anxiety, and stress were all contributing factors to risk-taking behaviors that may increase the need for HIV testing. These same mental health symptoms were present among a sample of individuals seeking HIV testing [24]. Also, those who were previously diagnosed with a mental illness were found more likely to have had prior HIV testing [29]. It is important to note that many studies in this area have been conducted with adult populations in the United States and Canada. Age and context may contribute to current findings. That is, mental health symptoms may be viewed differently by youth and/or by other individuals in low-to-middle income countries. Social acceptability of admitting to mental health concerns or even differing thresholds of tolerance may have had an impact on the significance of these factors in the current analysis. 

The marginally significant negative predictive effect of social support on intent to be tested for HIV found in this study is inconsistent with what is understood about the importance of social interaction in maintaining physical and mental health [15,17,19]. Cultural factors and stigma may help explain current findings. Given the stigma associated with HIV in Kenya, it is possible that fear of rejection by social groups may have served as a barrier to HIV testing intentions for participants in this sample [53]. That is, youth with a strong social support network may fear losing support if others find out they were tested for HIV given assumptions that might be made about promiscuity or other high risk behaviors.

**Figure 1:**
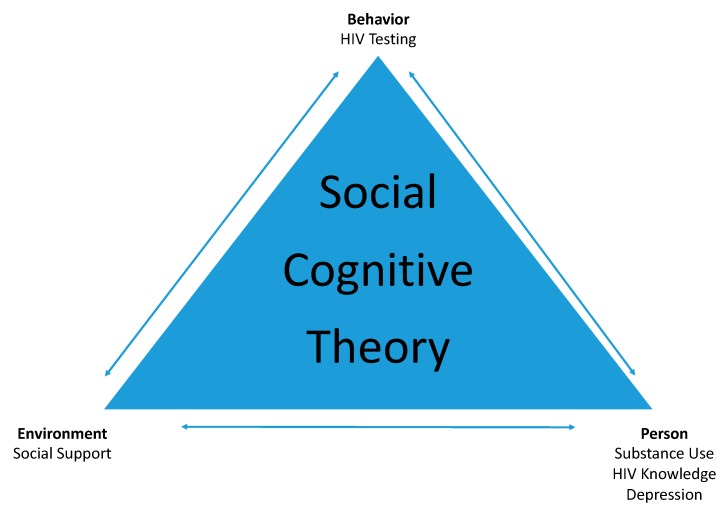
Social Cognitive Theory (SCT) Applied to HIV Testing.

While several of the hypothesized facilitators and barriers to HIV testing were not found to be significant in the current study (i.e., stigma, subjective well-being, anxiety, stress), findings do provide some support for the use of social cognitive theory to explain HIV testing behaviors among youth [34]. Specifically, social support—an environmental factor—and substance use, HIV knowledge, and depression—personal factors—were found to be predictors of HIV testing—a behavioral factor. See Figure 1. However, several of the personal factors hypothesized for the model were not significant among the study sample as described above. Specifically, projected stigma, anxiety, stress, and subjective well-being did not predict HIV testing in the current sample. With regard to stigma, it is important to note that HIV stigma was associated with HIV testing intentions in a simple regression, but lost significance when other variables were taken into account. This is likely due to multicollinearity among the predictors used in this study, which suggests that stigma does not uniquely explain HIV testing intentions when more powerful predictors are taken into consideration.

There were several limitations to this study. First, while the measures used in this study were established as reliable measures in the literature, estimates of internal consistency among our sample were moderate for the social support and substance use measures and low for the HIV knowledge and projected stigma measures. One explanation for this relates to the homogeneity of the sample, since all participants were part of the same culture and fell within a restricted age range. In addition, there are other measure-related factors that can affect alphas—the length of the measures, use of subscales, interrelatedness, and construct heterogeneity [54]. Alphas should not be considered the only indicator of reliability, since low alphas do not always indicate poor measures, and high alphas are not necessarily proof of a reliable measure [55]. 

Second, cultural differences and language barriers may have been limitations in this study. While questionnaires were translated into Swahili (the native language in Kenya) and offered in both English and Swahili, all participants chose to complete the English version; this is likely due to the fact that English is taught and used in educational settings in the region [40]. Thus, youth might be more comfortable reading and writing in English while they are more comfortable speaking in their native language of Swahili [40]. Nonetheless, it is possible that individual items on measures were misinterpreted or misunderstood due to the cultural context. 

Finally, the research design posed limitations. The use of existing program evaluation data resulted in limited control over the variables of interest in this study. For example, intent to test for HIV was the main outcome in this study. While several theories (e.g., theory of reasoned action and theory of planned behavior) suggest a relationship between intentions and behavior [56] and it is not uncommon for researchers to assess intentions as opposed to actual behavior in studies related to HIV testing [57,58], we do not know if intent to test resulted in future HIV testing among our participants. Also, the use of a correlational design is limiting with regard to the conclusions that can be made about relationships between variables. Further research implementing experimental designs is needed to identify causal relationships between personal and environmental factors, intent to test, and actual HIV testing.

## 5. Conclusions 

This research relied on existing data from a community-based participatory research project. Despite limitations, this study contributes to our knowledge regarding factors related to HIV testing intent among youth in Kenya. There is still much to be learned about youth at risk for HIV in Kenya and the behavioral, personal and environmental factors that may impact their healthcare. This study serves as a building block for future studies. 

Additional research is needed to explore factors affecting HIV testing among youth in low-to-middle income countries (LMICs). Post and follow-up data from the HIV SEERs Project may yield important information about the impact of a community-based participatory research project on HIV testing and treatment. Beyond SEERs, it is imperative that further research include larger and more representative samples and take into consideration language and cultural barriers. Additionally, intervention studies designed to improve rates of HIV testing among youth in LMICs will build upon the information collected in this and similar studies. 

## Figures and Tables

**Table 1:** Predicting HIV Testing Intentions: Logistic Regression Forward Selection—Final Model (*N* = 651).

Variable	*Exp(B)*	*b*	S.E.(*b*)	χ^2^	*p*
HIV knowledge	1.091	0.087	0.035	6.186	0.013
Substance use	1.413	0.346	0.128	7.334	0.007
Depression	0.981	−0.019	0.010	3.716	0.054
Social support	0.976	−0.024	0.012	3.682	0.055

*Note.* S.E. = standard error. Exp(B) = odds ratio.
